# Transcriptome-Wide N^6^-Methyladenosine (m^6^A) Profiling of Susceptible and Resistant Wheat Varieties Reveals the Involvement of Variety-Specific m^6^A Modification Involved in Virus-Host Interaction Pathways

**DOI:** 10.3389/fmicb.2021.656302

**Published:** 2021-05-26

**Authors:** Tian-ye Zhang, Zi-qiong Wang, Hai-chao Hu, Zhi-qing Chen, Peng Liu, Shi-qi Gao, Fan Zhang, Long He, Peng Jin, Miao-ze Xu, Jian-ping Chen, Jian Yang

**Affiliations:** ^1^College of Plant Protection, Nanjing Agricultural University, Nanjing, China; ^2^State Key Laboratory for Quality and Safety of Agro-Products, Institute of Plant Virology, Ningbo University, Ningbo, China

**Keywords:** wheat, m^6^A methylation, m^6^A-seq, RNA-seq, WYMV, plant-pathogen interacting, transcriptional regulation

## Abstract

N^6^-methyladenosine (m^6^A) methylation is the most prevalent internal modification of post-transcriptional modifications in mRNA, tRNA, miRNA, and long non-coding RNA in eukaryotes. m^6^A methylation has been proven to be involved in plant resistance to pathogens. However, there are no reports on wheat (*Triticum aestivum*) m^6^A transcriptome-wide map and its potential biological function in wheat resistance to wheat yellow mosaic virus (WYMV). To the best of our knowledge, this study is the first to determine the transcriptome-wide m^6^A profile of two wheat varieties with different resistances to WYMV. By analyzing m^6^A-sequencing (m^6^A-seq) data, we identified 25,752 common m^6^A peaks and 30,582 common m^6^A genes in two groups [WYMV-infected resistant wheat variety (WRV) and WYMV-infected sensitive wheat variety (WSV)], and all these peaks were mainly enriched in 3′ untranslated regions and stop codons of coding sequences. Gene Ontology analysis of m^6^A-seq and RNA-sequencing data revealed that genes that showed significant changes in both m^6^A and mRNA levels were associated with plant defense responses. Kyoto Encyclopedia of Genes and Genomes analysis revealed that these selected genes were enriched in the plant–pathogen interaction pathway. We further verified these changes in m^6^A and mRNA levels through gene-specific m^6^A real-time quantitative PCR (RT-qPCR) and normal RT-qPCR. This study highlights the role of m^6^A methylation in wheat resistance to WYMV, providing a solid basis for the potential functional role of m^6^A RNA methylation in wheat resistance to infection by RNA viruses.

## Introduction

RNA molecules are crucial in all living organisms, playing significant roles in passing genetic information and regulating several biological processes (Fu et al., 2014). The structure of RNA transcripts may be adjusted through complex chemical modifications to perform specific molecular functions. To date, more than 100 types of RNA modifications have been identified. Among these post-transcriptional modifications, the most common RNA modifications are N^6^-methyladenosine (m^6^A), N^5^-methylcytosine, and N1-mythylcytosine (Cantara et al., 2011; Wei et al., 2017). However, m^6^A, which was first detected in the 1970s, is the most prevalent internal mRNA modification in eukaryotes, including mammals, plants, and yeast, as well as viruses (Wei et al., 1975). The m^6^A modification is induced by some m^6^A methyltransferases, called m^6^A “writers,” such as methyltransferase-like 3 and 14 (METTL3 and METTL14), as well as Wilms tumor 1-associated protein (WTAP), in mammalian cells (Fu et al., 2014; Liu et al., 2014; Ping et al., 2014). Furthermore, the m^6^A modification can be dynamically regulated by m^6^A demethylases, called m^6^A erasers, including fat mass and obesity-associated protein (FTO) and ALKB homolog 5 (ALKBH5), to maintain the m^6^A modification in a dynamic balance (Jia et al., 2011; Zheng et al., 2013; Liu et al., 2014). Another series of m^6^A-binding proteins (readers), such as YTHDF2 and YTHDF3, which belong to the YT521-B homology domain family, can bind specifically to m^6^A-modified cellular RNAs to carry out the biological function of methylation (Zhang et al., 2010; Dominissini et al., 2012; Xu et al., 2014, 2015). In general, the m^6^A modification can efficiently control gene expression, plant development, and physiological processes.

Through continuous research on the m^6^A modification, the most substantially enriched motif—RRACH (R = A/G, H = A/C/U)—has been authenticated through transcriptome-wide mapping of m^6^A in m^6^A peaks of all eukaryotes analyzed to date, consistent with early biochemical studies of eukaryote mRNA (Wei et al., 1976; Dimock and Stoltzfus, 1977; Schibler et al., 1977; Canaani et al., 1979; Nichols and Welder, 1981). Recently, another plant-specific consensus motif URUAY (R = A/G, Y = C/U) was identified using the m^6^A reader, ECT2 protein (Wei et al., 2018). The regulatory machinery of the m^6^A modification is post-transcriptionally assembled by the conserved methylation-related protease at the conserved consensus sequence, RRACH or URUAY. In plants, m^6^A is generally enriched in stop codons, start codons, and 3′-untranslated regions (UTRs), especially at the 3′-end of coding sequences (CDS) and the front end of 3′-UTRs (Dominissini et al., 2013; Schwartz et al., 2013, 2014). An increasing number of studies have shown that m^6^A can affect or regulate RNA expression and transcription in living cells under stress. For example, translation initiation for mammalian stress-responsive genes were promoted through direct binding of 5′-UTR m^6^A to eukaryotic initiation factor 3 (eIF3) and recruiting the 43S ribosomal complex in a cap-independent manner under the heat stress (Meyer et al., 2015). The intensity of 30% of m^6^A peaks is altered by ultraviolet light, heat shock, or interferon-gamma, thereby affecting gene expression and splicing (Dominissini et al., 2012). In METTL3 mutants, the translation of mRNAs with m^6^A modifications in the 5′-UTR is significantly reduced, and thus 5′-UTR m^6^A affects translation efficiency in cells (Meyer and Jaffrey, 2017). Increasing evidence indicates that m^6^A is also involved in regulating responses to abiotic stresses. In tobacco mosaic virus (TMV)-infected *Nicotiana tabacum* plants, the m^6^A level decreased, whereas ALKBH5-dependent m^6^A demethylation was promoted (Li et al., 2018). Indeed, alfalfa mosaic virus (AMV) infection induces the erasure of m^6^A in the viral genome and promotes systemic infection (Martínez-Pérez et al., 2017). With the development of high-throughput assays, profiling the m^6^A modification pattern on a transcriptome-wide scale has become possible. Currently, m^6^A-sequencing (m^6^A-seq) has been further developed, and transcriptome-wide mapping of m^6^A is now possible through m^6^A-RNA immunoprecipitation, followed by high-throughput sequencing (Dominissini et al., 2012; Meyer et al., 2012).

Wheat RNA viruses, especially wheat yellow mosaic virus (WYMV), cause a severe reduction in production in the major wheat-growing areas in China (Han et al., 2000; Liu et al., 2005). WYMV is one of the main pathogens of wheat soil-borne mosaic disease and belongs to the genus *Bymovirus* (Potyviridae) (Zhang et al., 2019). The WYMV genome contains two positive single RNA strands, RNA1 (7.5 kb) and RNA2 (3.6 kb) (Zhang et al., 2019). Wheat infected by WYMV show mosaic or yellow-striped leaves and plant stunting, and grain yield is reduced by 20–70% in some severely affected fields (Chen et al., 2014). In RNA viruses, including hepatitis C virus (HCV), Zika virus, dengue virus, and West Nile virus, the m^6^A modification has been identified in their genome (Gokhale et al., 2016; Courtney et al., 2017). Nevertheless, there have been very few studies on transcriptome-wide m^6^A methylome profiling of RNA viruses, especially WYMV, in different varieties of wheat. To the best of our knowledge, the present study is the first to perform transcriptome-wide m^6^A profiling in a WYMV-infected resistant wheat variety (yannong 999) (hereinafter referred to as WRV) and a WYMV-infected sensitive wheat variety(yannong 24) (hereinafter referred to as WSV), and found a significant variation in the m^6^A modification patterns between the resistant and susceptible varieties. Furthermore, different m^6^A RNA modifications in the two varieties demonstrated regulation of gene expression and pathogen-plant interaction-related pathways. Our study provides a basis for a comprehensive understanding of the roles of m^6^A modification in the molecular mechanisms underlying the interaction between wheat host and RNA viruses, especially WYMV.

## Materials and Methods

### Plant Materials

To obtain sufficient total RNA for IP of m6A-containing mRNA, ∼30 reviving stage plants each of yannong 999 and yannong 24 wheat infected or un-infected with WYMV were collected from a diseased nursery in Yantai city, Shandong province, China, respectively. WYMV-infected yannong 24 plants with typical yellow mosaic symptoms on the leaves, show stunted spring growth and reduced tillering. WYMV-infected yannong 999 plants show normal phenotypes without yellow mosaic symptoms (Supplementary Figure 3). Each wheat plants groups were equally divided into three mixed samples as three biological repeats respectively for RNA extraction and m^6^A IP sequence. These samples Stored at −80°C for RNA extraction and IP-qPCR and m^6^A IP sequence.

### RNA Extraction and Fragmentation

Wheat leaves were collected, frozen, and stored at −80°C until use. Total RNA was extracted from plants using Trizol reagent (Invitrogen). NanoDrop ND-1000 (NanoDrop, Wilmington, DE, United States) was used to quantify the amount and purity of RNA in each sample. RNA integrity was investigated using a Bioanalyzer 2100 (Agilent, CA, United States) with RIN number >7.0, and further confirmed through electrophoresis with denaturing agarose gel. Then, Dynabeads Oligo (dT) 25–61005 (Thermo Fisher Scientific, CA, United States) was used to purify the poly (A) RNA from 150 μg total RNA. Finally, the poly(A) RNA was randomly fragmented into small pieces using a Magnesium RNA Fragmentation Module (cat. e6150, United States).

### m^6^A IP and Library Construction

The total RNA was fragmented and then incubated with m^6^A-specific antibody (No. 202003, Synaptic Systems, Germany) in IP buffer (50 mM Tris–HCl, 750 mM NaCl, and 0.5% Igepal CA-630) for 2 h at 4°C. Then, the IP RNA was reverse-transcribed to cDNA, and U-labeled second-stranded DNAs were synthesized using cDNA with E. coli DNA polymerase I, RNase H (NEB, United States), and dUTP Solution (Thermo Fisher Scientific, United States). Then, the strands were prepared with A-base and ligated to the indexed adapters containing a T-base overhang for ligating the adapter to the A-tailed fragmented DNA. AMPureXP beads were used to screen fragments of the right size. The U-labeled second-stranded DNAs treated with heat-labile UDG enzyme (NEB, United States) were next amplified by PCR to generate a cDNA library with an average insert size of 300 ± 50 bp. Finally, 2 × 150 bp paired-end sequencing (PE150) was performed on an Illumina Novaseq^TM^ 6000 (LC-BioTechnology Co., Ltd., Hangzhou, China), following the manufacturer’s recommended protocol.

### Data Analysis

The raw data were processed by the online FASTP software^1^ to remove reads with adaptor contamination, low quality bases, and bases with undefined default parameters. HISAT2, an online software^2^ to map reads to the reference genome of *Triticum aestivum* (Version: IWGSC v1.0). Mapped reads of IP and input data were used for analysis using the R package exomePeak document^3^ then m6A intensity was visualized using Integrative Genomics Viewer (IGV) software^4^. MEME and HOMER^5^^6^ were used for motif analysis. Called peaks were annotated by intersection with gene architecture using the R package ChIP seeker. The expression levels of all mRNAs from the input libraries were determined using StringTie^7^ by calculating FPKM [total exon fragment/mapped reads (millions) × exon length (kB)]. Differentially expressed genes were screened out using the criteria: fold change >1.5 or fold change <0.5, and *p*-value < 0.05 by the R package edgeR^8^.

### Gene-Specific m^6^A qPCR and Normal qPCR

Total RNA was extracted and fragmented into 300-nucleotide-long fragments and incubated with anti-m^6^A antibody-coupled beads, while a portion of fragmented RNA was used as an input control. After ethanol precipitation, the input RNA and immunoprecipitated RNA were eluted from the beads. Both input control and m^6^A-IP samples were subjected to real-time quantitative PCR (RT-qPCR) with gene-specific primers. RT-PCR analysis was performed using an ABI Q5 Sequence Detection System (Applied Biosystems, CA, United States) with AceQ qPCR SYBR Green Master Mix (Vazyme, Nanjing, Jiangsu, China). At least, three biological replicates, with three technical replicates, were used for each assay. The *T. aestivum* cell division cycle (*TaCDC*) gene (Accession Number: XM_020313450) was used as the internal reference gene for analysis to calculate the fold changes in gene expression. The fold changes were calculated using the 2^–Δ^ ^Δ^ ^*C(t)*^ method (Livak and Schmittgen, 2002). The calculation of specific mRNA fragment m^6^A levels was performed as previously described (Miao et al., 2020). In brief, relative enrichment of each fragment was calculated by first normalizing both the number of target cDNA fragment and the internal control, after which the value for the immunoprecipitated sample and the input was also normalized. And the input was used as the internal control for analysis to calculate the fold changes using the 2^–ΔΔ*C(t)*^. Statistical analyses were performed done using the Student’s *t*-test. Asterisks indicate a significant difference when compared to the control. ^∗^*p* < 0.05; ^∗∗^*p* < 0.01.

## Results

### Transcriptome-Wide Detection of the m^6^A Modification in WYMV-Infected Resistant Wheat Variety and WYMV-Infected Sensitive Wheat Variety

Leaf tissues from the WRV and the WSV were collected. Firstly, the accumulation of WYMV was detected in the total RNA extracted from WRV and WSV leaves by qRT-PCR assay using *CP*-specific primers. The results assay showed that the accumulation of WYMV was significantly increased in WSV than that in WRV (Supplementary Figure 1). And then these samples were used for transcriptome-wide m^6^A-seq and RNA-sequencing (RNA-seq) using the Illumina Novaseq^TM^ 6000. A total of 129–139 million reads were acquired from the m6A-seq dataset, and 124–136 million reads were obtained from the RNA-seq dataset. After statistical analysis and quality control, there were still 100–135 million and 123–133 million valid reads in the m^6^A IP-seq and RNA-seq datasets, respectively. Indeed, the reads with a sequencing error rate <0.1% were more than 92% (Q30 > 92%), indicating that our data were clean (Supplementary Table 1). According to the wheat (*T. aestivum*) (Version: IWGSC v1.0) reference genome, more than 90% of the reads in the m^6^A IP-seq dataset were mapped. Moreover, almost 70% of the valid reads were uniquely mapped reads, similar to the results of a previous study on rice (Supplementary Table 2; Li et al., 2014).

**FIGURE 1:**
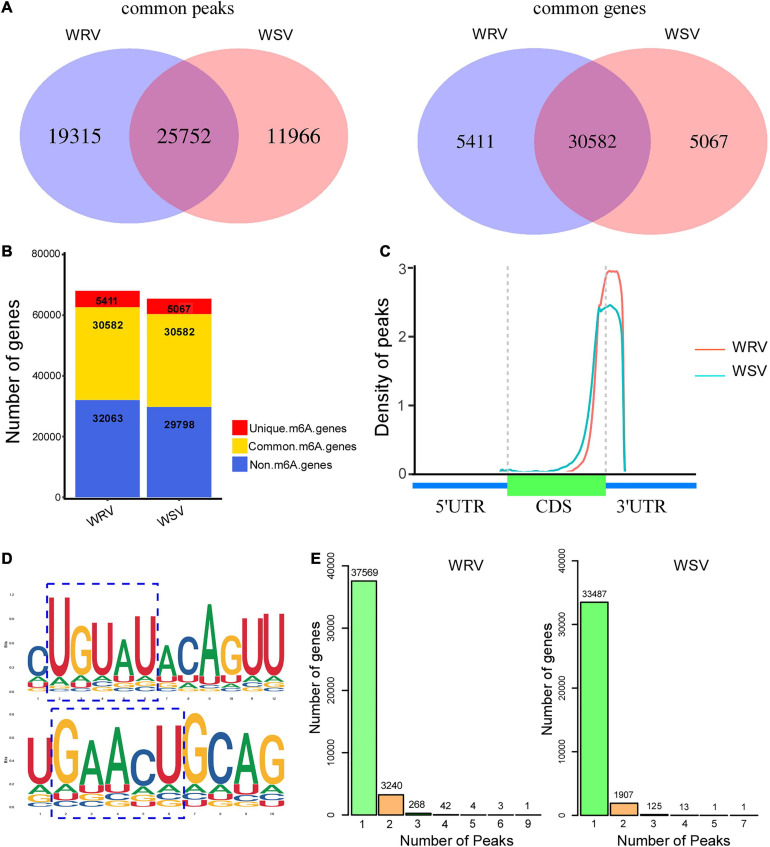
Overview of m^6^A methylome in two varieties of wheat and analysis of m^6^A peaks. **(A)** *Left*: numbers of WRV unique peaks and WSV unique and common peaks. *Right*: numbers of these peaks represent genes of the two groups. **(B)** Numbers of m^6^A-modified genes identified in m^6^A-seq data. **(C)** Density of m^6^A peaks along the wheat genome, which was divided into three non-overlapping regions, including 5′-UTR, CDS, and 3′-UTR. **(D)** Two top m^6^A motifs were enriched in all identified m^6^A peaks, RRACH and UGUAY (R is A/G, H is A/C/U, and Y is C/U). The blue dotted line box represented the conserved m^6^A motifs. **(E)** Numbers of m^6^A methylated genes with different numbers of m^6^A peaks in the two groups. WRV, WYMV-infected resistant wheat variety; WSV, WYMV-infected sensitive wheat variety.

The total m^6^A peaks (actually identified as m^6^A modification sites) of the two groups were identified by comparing the mapped reads of IP and input [two sequencing libraries, named m^6^A-seq library (IP) and RNA-seq library (Input)] using the R package, exomePeak. There were 45,067 m^6^A peaks in the WRV group and 37,718 m^6^A peaks in the WSV group. Moreover, the m^6^A peaks in the WRV and WSV groups represented transcripts of 35,993 and 35,649 genes, respectively (Figure 1A). Through the Venn diagram method, we found 25,752 common peaks representing 30,582 common genes that were m^6^A-modified in both groups (Figure 1A). Furthermore, there were 5411 genes and 5067 genes that underwent m^6^A modification in the WRV and WSV groups, respectively (Figure 1B). The distribution of m^6^A peaks in the whole transcriptome for these two groups was investigated according to gene annotations in the reference genome. The result showed that the vast majority of m^6^A peaks were centrally located near 3′-UTRs and stop codons of CDS (Figure 1C). The unique m^6^A peak distribution in both WRV and WSV groups are shown in Supplementary Figure 2. HOMER, a motif analysis software, was used to identify reliable motifs in the peak region in each group of samples. Two high consensus motifs, UGUAY and GAACU, were identified in the m^6^A peaks (Figure 1D), consistent with previous studies (Fu et al., 2014; Miao et al., 2020). Indeed, we analyzed the numbers of m^6^A-modified sites per m^6^A-modified gene and found that more than 99% of the genes (A: 37569/41127, B:33487/35534) had only one or two m^6^A-modified sites. However, genes with two m^6^A-modified sites in the WRV group (7.8%) were more than those in the WSV group (5.3%), and the WRV group unique m^6^A genes had more methylated sites than those of the WSV group (Figure 1E). In summary, these data indicated significant differences in the m^6^A modification patterns between the WRV and WSV groups.

### Differentially Methylated Genes Are Enriched in Plant–Pathogen Interaction-Related Pathways

**FIGURE 2:**
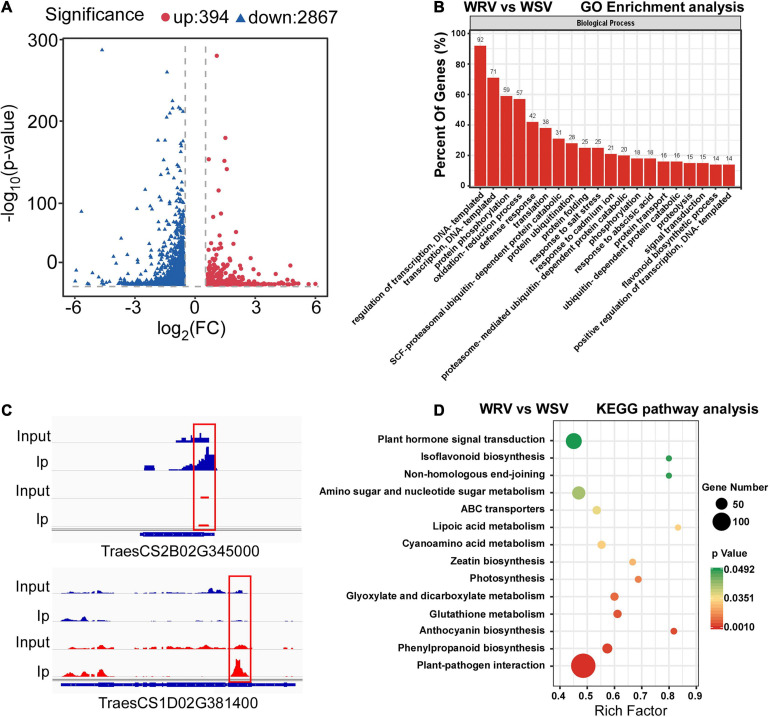
Global m^6^A modification changes in two WYMV-infected varieties and functional analysis of genes with m^6^A. **(A)** Identification of 394 hyper-methylated and 2867 hypo-methylated m^6^A peaks in the WRV group compared with the WSV group that showed a significant increase or decrease in m^6^A methylation (fold change >1.5, *p* < 0.05). **(B)** Examples of hyper-methylated and hypo-methylated genes with differential m^6^A peaks. The blue peaks represent the IP and Input reads from the WRV group, and the red peaks represent the IP and Input reads from the WSV group. The red boxes represent m^6^A peaks. **(C)** Gene Ontology analysis of biological processes of differentially methylated genes (DMGs). The vertical axis represents the proportion of DMGs enriched in each pathway. **(D)** KEGG pathway enrichment analysis of the DMGs. The horizontal axis represents the rich factor of each pathway.

To determine the differentially methylated peaks between the WRV and WSV groups, the m^6^A-seq dataset of the WRV group was compared to that of the WSV group, and then 3261 differentially methylated sites were screened out, including 394 hyper-methylated m^6^A sites and 2867 hypo-methylated m^6^A sites (fold change >1.5, *p* < 0.05) (Figure 2A). Furthermore, we randomly selected two differentially methylated sites from the hyper-methylated and hypo-methylated site database. The genes corresponding to these two differentially methylated sites (hyper-methylated: TraesCS2B02G345000, hypo-methylated: TraesCS1D02G381400) showed altered methylation intensity, using the IGV software (Figure 2B).

To investigate the potential biological processes in which the m^6^A-modified genes are involved, Gene Ontology (GO) enrichment analysis of these differentially methylated genes (DMGs) was performed. The results showed that the DMGs were mainly enriched in DNA transcription, protein post-translational modification (PTM), stress response including DNA-templated transcription, protein phosphorylation, ubiquitin-dependent protein catabolic process and defense response, respectively (Figure 2C). To further predict the functions of these m^6^A-modified genes, Kyoto Encyclopedia of Genes and Genomes (KEGG) pathway analysis was performed, revealing that the majority of the DMGs were enriched in plant hormone signal transduction and plant–pathogen interaction pathways (Figure 2D). Collectively, these results demonstrated that the m^6^A modification may be involved in a variety of biological pathways, such as PTM and defense response, while being closely related to plant host and pathogen interaction.

### Differentially Expressed Gene Analysis by RNA-Seq

**FIGURE 3:**
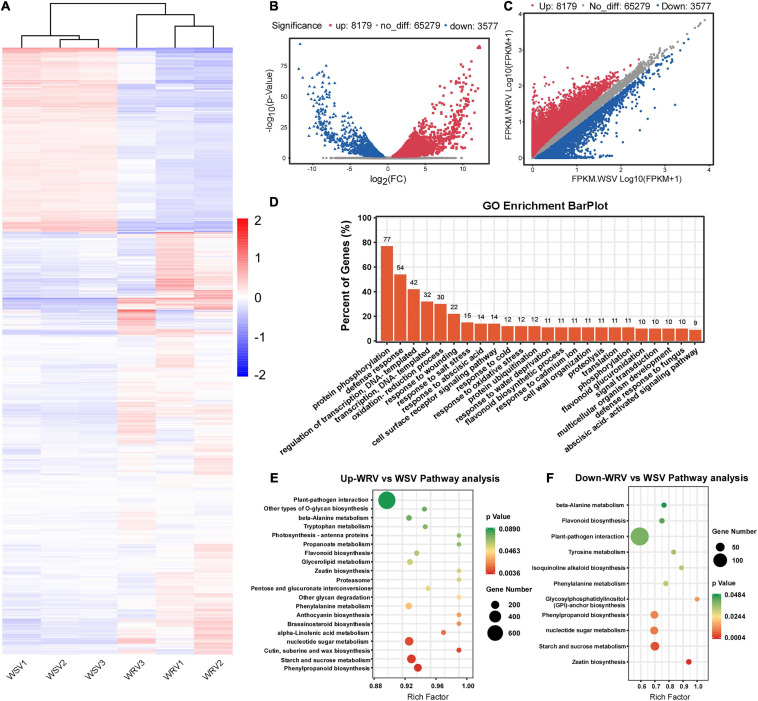
Overview of differentially expressed genes in the WRV group compared with the WSV group by RNA-seq and functional analysis of differentially expressed genes. **(A)** Heat map of RNA-seq data of WRV and WSV samples. Rows, mRNAs; columns, WRV samples and WSV samples. Red, white, and blue represent upregulation, unchanged expression, and downregulation of mRNA levels, respectively. **(B)** Volcano analysis of differentially expressed genes. **(C)** Scatter plot of differentially expressed genes. FPKM, fragments per kilobase of exon model per million mapped reads. **(D)** Gene Ontology analysis of the biological processes of differentially expressed genes. The vertical axis represents the proportion of differentially expressed genes enriched in each pathway. **(E,F)** KEGG pathway enrichment analysis of the upregulated and downregulated genes, respectively. The horizontal axis represents the rich factor of each pathway.

To explore the potential relationship between m^6^A modification and gene expression, differentially expressed gene analysis was performed using the input sequencing data. Through hierarchical clustering of the RNA-seq data, we found obvious differential global mRNA expression patterns between the WRV and WSV groups (Figure 3A). We then screened the RNA-seq database and identified 8179 upregulated genes and 3577 downregulated genes in the WRV samples compared to the WSV samples (fold change >1.5, *p* < 0.05) (Figures 3B,C). Subsequently, these selected differentially expressed genes were used for GO enrichment and KEGG pathway analyses. The results showed that more than 70% of the differentially expressed genes were enriched in protein PTMs, such as protein phosphorylation and ubiquitination processes, while 54% were enriched in defense responses, including defense against fungi (Figure 3D). To further investigate which pathways the upregulated or downregulated genes participate in, these two types of genes were used for KEGG pathway analysis. The results revealed that the upregulated and downregulated genes were both enriched in the plant–pathogen interaction pathway, consistent with the pathogenic mechanism of the virus and the immune mechanism of the plant (Figures 3E,F; Mandadi and Scholthof, 2013; Wang, 2015). Therefore, we speculated that these differentially expressed genes may be affected by virus infection and may be involved in plant immunity.

### Association Analysis Between m^6^A-Seq and RNA-Seq

**FIGURE 4:**
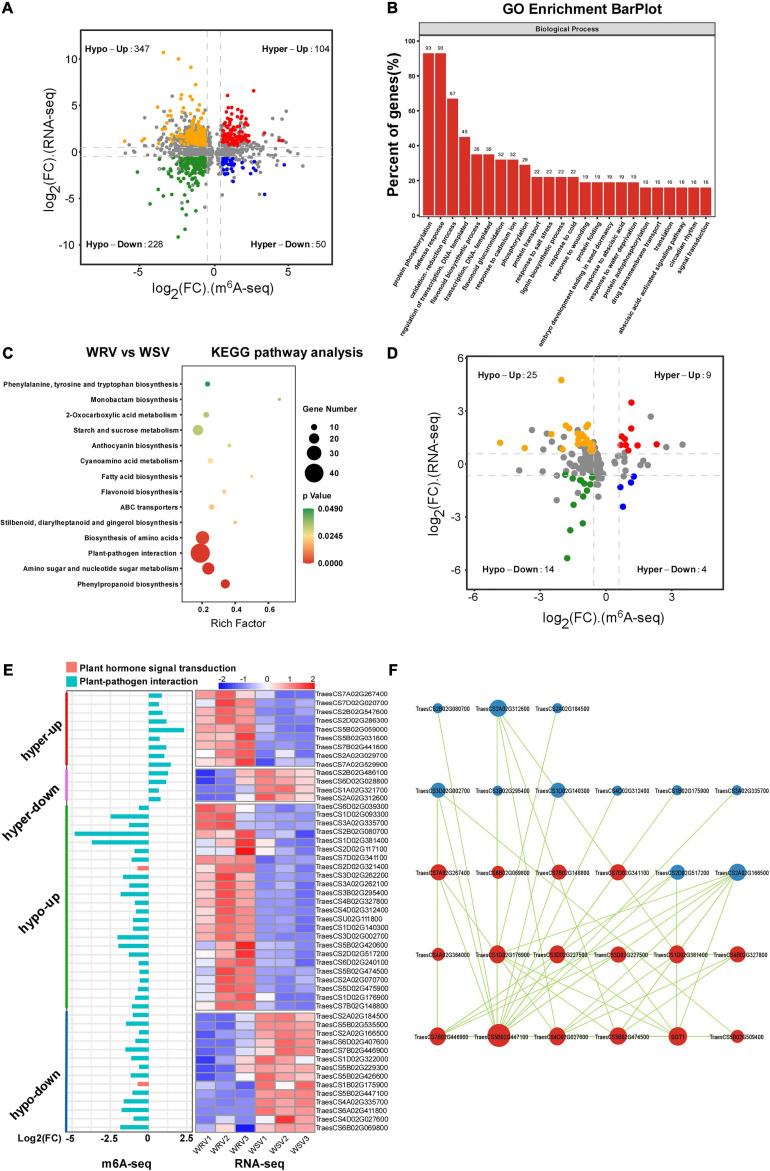
Conjoint analysis of m^6^A-seq and RNA-seq data. **(A)** A four-image map to analyze the relationship between differential genes and differential peaks and to screen out genes with a significant change in both m^6^A and mRNA levels in WRV samples compared with WSV samples (fold change >1.5, *p* < 0.05). **(B)** Gene Ontology analysis of biological processes of genes with a significant change in both m^6^A and mRNA levels. The vertical axis represents the proportion of selected genes enriched in each pathway. **(C)** KEGG pathway enrichment analysis of the genes with a significant change in both m^6^A and mRNA levels. The horizontal axis represents the rich factor of each pathway. **(D)** Distribution of genes with a significant change in both m^6^A and mRNA levels that were enriched in the plant–pathogen interaction pathway and plant hormone signal transduction pathway (fold change >1.5, *p* < 0.05). **(E)** Heat map and bar diagram of “hyper-up,” “hyper-down,” “hypo-up,” and “hypo-down” genes represented in **D**. Left, m^6^A-seq; right, RNA-seq. The red column represents genes enriched in both pathways, and blue column represents genes enriched only in plant–pathogen interaction pathway. Red, white, and blue represent upregulation, unchanged expression, and downregulation of mRNA level, respectively. **(F)** Graph of the protein interaction network of abnormally m^6^A-modified genes enriched in the plant–pathogen interaction pathway generated with the OmicStudio tool at https://www.omicstudio.cn/tool/56. Red and blue circles represent the source and target genes, respectively. The size of the circle represents the complexity of the interaction.

Previous studies have reported that m^6^A RNA modifications are related to gene expression (Luo et al., 2014; Lockhart, 2018). Therefore, we performed a cross-analysis of the m^6^A-seq and RNA-seq data (fold change >1.5, *p* < 0.05), and the results were displayed using a four-quadrant diagram, with the following four parts: hypo-up and hypo-down, and hyper-up and hyper-down, representing hypo/hyper m^6^A modifications causing gene expression up/down regulation, respectively. There were 729 differentially genes, among which 347 genes belonged to the hypo-up, 104 genes to the hyper-up, 228 genes to the hypo-down, and 50 genes to the hyper-down quadrants (Figure 4A). Furthermore, these selected genes were used for GO enrichment and KEGG pathway analyses (Figures 4B,C). Interestingly, 93% of the selected genes were related to plant defense response, while many of them were enriched in plant–pathogen interaction pathways, and some were involved in both plant–pathogen interaction and plant hormone signal transduction pathways. Next, the genes enriched in the two pathways were chosen for the four-quadrant diagram, and the majority of them had hypo m^6^A modifications. Among these hypo m^6^A-modified genes, 25 were upregulated at the mRNA level and 14 were downregulated (Figure 4D). In addition, 52 genes associated with these two pathways showed a significant difference in both m^6^A RNA methylation levels and mRNA expression levels (Table 1). The relationship between m^6^A RNA methylation level and mRNA expression level is presented more intuitively in Figure 4E, including the corresponding gene numbers. To further explore the interactions between these selected genes, we carried out protein interaction network analysis using the OmicStudio tool (Figure 4F). Many proteins were predicted to interact with SGT1 (TraesCS3D02G227500) protein, which has been reported to be associated with plant resistance to pathogens (Wang et al., 2015). Therefore, these genes and related pathways may have great significance in the protein–protein interaction network and molecular events in the viral infection process.

**TABLE 1:** List of 52 genes that exhibit a significant change in both m6A level and mRNA transcript abundance in WRV compared with WSV.

Gene name	Pattern	Chromosome	m6A level change					mRNA level change		
			Peak annotation	Peak start	Peak end	Fold change	*p*-value	Strand	Fold change	*p*-value
TraesCS2A02G029700	Hyper-up	chr2A	3′ UTR	13,251,654	13,251,832	2.0279	0.00302	+	1.7010	0.04004
TraesCS2B02G547600	Hyper-up	chr2B	3′ UTR	744,166,622	744,167,130	1.8738	0.00030	−	2.1033	0.00168
TraesCS2D02G286300	Hyper-up	chr2D	3′ UTR	365,779,571	365,782,391	2.2346	0.03548	−	11.1247	0.00000
TraesCS5B02G031600	Hyper-up	chr5B	3′ UTR	34,722,046	34,722,525	1.6426	0.00832	+	2.9500	0.00030
TraesCS5B02G059000	Hyper-up	chr5B	3′ UTR	64,731,218	64,732,506	4.9588	0.00000	+	2.1612	0.00161
TraesCS7A02G267400	Hyper-up	chr7A	3′ UTR	270,761,429	270,761,817	1.8100	0.04677	+	2.6770	0.01667
TraesCS7A02G529900	Hyper-up	chr7A	3′ UTR	709,404,773	709,405,192	2.7132	0.00000	+	2.0710	0.00971
TraesCS7B02G441600	Hyper-up	chr7B	3′ UTR	706,808,371	706,808,639	2.2191	0.00060	+	4.0297	0.00000
TraesCS7D02G020700	Hyper-up	chr7D	3′ UTR	9,303,950	9,304,570	1.5867	0.00417	+	2.1060	0.00767
TraesCS1A02G321700	Hyper-down	chr1A	3′ UTR	512,651,452	512,651,749	1.5768	0.02692	+	0.4032	0.00597
TraesCS2A02G312600	Hyper-down	chr2A	3′ UTR	537,252,891	537,253,188	1.7088	0.00000	+	0.1872	0.00000
TraesCS2B02G486100	Hyper-down	chr2B	3′ UTR	683,048,765	683,049,780	2.4116	0.00000	+	0.6119	0.03105
TraesCS6D02G028800	Hyper-down	chr6D	Exon	11,091,468	11,091,677	2.2038	0.01122	+	0.4843	0.02553
TraesCS1D02G176900	Hypo-up	chr1D	3′ UTR	251,985,355	251,985,654	0.5411	0.00045	+	2.6136	0.01048
TraesCS1D02G093300	Hypo-up	chr1D	3′ UTR	79,132,968	79,133,298	0.1780	0.00006	−	3.2244	0.00035
TraesCS1D02G140300	Hypo-up	chr1D	3′ UTR	193,360,790	193,361,027	0.4931	0.00000	−	2.9749	0.00003
TraesCS1D02G381400	Hypo-up	chr1D	Exon	456,287,493	456,287,792	0.0764	0.00000	−	1.8709	0.04330
TraesCS2A02G070700	Hypo-up	chr2A	Exon	31,177,089	31,177,459	0.6666	0.00107	+	1.7185	0.03833
TraesCS2B02G080700	Hypo-up	chr2B	Exon	45,040,125	45,040,185	0.0352	0.00000	+	2.2978	0.00247
TraesCS2D02G117100	Hypo-up	chr2D	3′ UTR	66,759,926	66,762,104	0.5263	0.00000	−	2.2422	0.03075
TraesCS2D02G321400	Hypo-up	chr2D	3′ UTR	412,833,158	412,833,755	0.6046	0.00000	+	1.7155	0.03374
TraesCS2D02G517200	Hypo-up	chr2D	3′ UTR	608,200,516	608,200,931	0.4090	0.00000	+	3.2992	0.00005
TraesCS3A02G262100	Hypo-up	chr3A	Exon	485,011,350	485,011,560	0.4175	0.00000	+	2.6098	0.00011
TraesCS3A02G335700	Hypo-up	chr3A	Exon	581,851,280	581,851,429	0.4118	0.00076	+	2.1840	0.04323
TraesCS3B02G295400	Hypo-up	chr3B	Exon	474,028,215	474,028,365	0.2813	0.00000	+	4.5309	0.00000
TraesCS3D02G262200	Hypo-up	chr3D	Exon	363,868,307	363,868,487	0.3121	0.00000	+	4.0562	0.00000
TraesCS3D02G002700	Hypo-up	chr3D	3′ UTR	1,252,991	1,253,291	0.2432	0.00195	+	26.8803	0.00000
TraesCS4B02G327800	Hypo-up	chr4B	3′ UTR	618,588,905	618,589,324	0.5363	0.00000	+	4.2774	0.00000
TraesCS4D02G312400	Hypo-up	chr4D	3′ UTR	478,267,912	478,268,332	0.5668	0.00000	−	4.6907	0.00000
TraesCS5B02G420600	Hypo-up	chr5B	3′ UTR	596,068,402	596,068,492	0.2535	0.00003	−	1.7805	0.03731
TraesCS5B02G474500	Hypo-up	chr5B	3′ UTR	647,940,529	647,940,947	0.6588	0.00000	−	2.1264	0.00609
TraesCS5D02G475900	Hypo-up	chr5D	3′ UTR	515,114,772	515,115,162	0.6134	0.00000	+	2.0456	0.00569
TraesCS6D02G039300	Hypo-up	chr6D	3′ UTR	16,199,065	16,199,595	0.6430	0.00000	+	1.9658	0.02814
TraesCS6D02G240100	Hypo-up	chr6D	3′ UTR	341,198,252	341,198,640	0.6360	0.00000	+	2.1286	0.00701
TraesCS7B02G148800	Hypo-up	chr7B	Exon	196,385,349	196,385,469	0.4698	0.00000	+	2.9533	0.00011
TraesCS7D02G341100	Hypo-up	chr7D	3′ UTR	436,954,924	436,955,825	0.4633	0.00035	+	3.2187	0.00035
TraesCSU02G111800	Hypo-up	chrUn	3′ UTR	95,997,036	95,997,514	0.6164	0.00000	+	2.2411	0.00102
TraesCSU02G111800	Hypo-up	chrUn	Exon	95,999,716	95,999,835	0.4897	0.00000	+	2.2411	0.00102
TraesCS1B02G175900	Hypo-down	chr1B	3′ UTR	317,422,653	317,423,775	0.6063	0.00525	+	0.4490	0.01475
TraesCS1D02G322000	Hypo-down	chr1D	3′ UTR	415,286,643	415,286,971	0.4538	0.00008	+	0.5257	0.04888
TraesCS2A02G184500	Hypo-down	chr2A	3′ UTR	144,851,567	144,854,464	0.4863	0.00000	+	0.2794	0.00000
TraesCS2A02G166500	Hypo-down	chr2A	Exon	119,011,088	119,012,124	0.6462	0.00000	+	0.6063	0.00802
TraesCS4A02G335700	Hypo-down	chr4A	3′ UTR	618,174,864	618,175,725	0.3231	0.04266	+	0.0744	0.00000
TraesCS4D02G027600	Hypo-down	chr4D	3′ UTR	12,411,599	12,412,107	0.5024	0.01122	−	0.4645	0.03711
TraesCS5B02G229300	Hypo-down	chr5B	3′ UTR	406,175,027	406,178,578	0.6457	0.00955	+	0.5879	0.02900
TraesCS5B02G535500	Hypo-down	chr5B	Exon	691,493,142	691,504,451	0.3585	0.00617	−	0.2018	0.00000
TraesCS5B02G426600	Hypo-down	chr5B	3′ UTR	602,340,150	602,340,419	0.4414	0.00081	−	0.5310	0.02180
TraesCS5B02G447100	Hypo-down	chr5B	3′ UTR	618,253,806	618,254,546	0.4796	0.00025	−	0.0974	0.00000
TraesCS6A02G411800	Hypo-down	chr6A	3′ UTR	613,964,281	613,964,788	0.2932	0.00263	+	0.0248	0.00000
TraesCS6B02G069800	Hypo-down	chr6B	Exon	46,886,313	46,886,372	0.2755	0.01259	+	0.6555	0.04803
TraesCS6D02G407600	Hypo-down	chr6D	3′ UTR	473,094,040	473,094,309	0.5479	0.02399	+	0.3521	0.00003
TraesCS7B02G446900	Hypo-down	chr7B	Exon	709,608,585	709,608,794	0.3487	0.00000	+	0.5661	0.00806

### Gene-Specific m^6^A RT-qPCR Assay and Normal RT-qPCR

**FIGURE 5:**
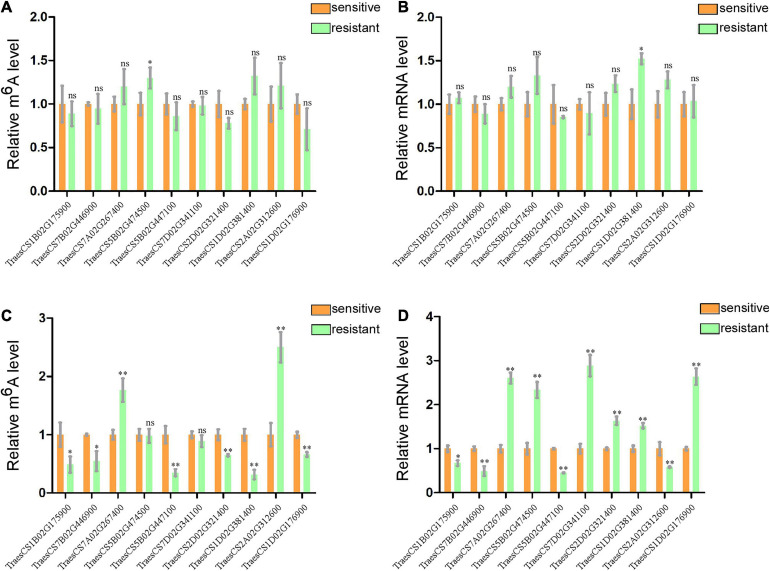
Gene-specific m6A/normal qPCR assays. **(A)** m^6^A IP qPCR validation of m6A methylation level of 10 randomly selected genes from Table 1 in two healthy wheat variety plants. **(B)** Relative mRNA levels of the 10 genes were detected *via* real-time qPCR in two healthy group plants. **(C)** m6A methylation level of 10 randomly selected genes in WRV and WSV group respectively. **(D)** Relative mRNA expression level of 10 genes in WRV and WSV group plants. The levels of the 10 genes in the sensitive variety group were normalized to 1. Statistical analyses were performed using the Student’s *t*-test. Asterisks indicate a significant difference when compared to the control. *p < 0.05; **p < 0.01.

To further confirm the results of our m^6^A-seq data, eight genes (TraesCS7B02G446900, TraesCS7A02G267400, TraesCS5B02G474500, TraesCS5B02G447100, TraesCS7 D02G341100, TraesCS1D02G381400, TraesCS2A02G312600, and TraesCS1D02G176900) among the central proteins shown in the protein interaction network with complicated interaction relationship, and two gene (TraesCS1B02G175900 and TraesCS2D02G321400) related to both plant–pathogen interaction and plant hormone signal transduction pathways were randomly selected for gene-specific m^6^A qPCR and normal RT-qPCR. To eliminate the effect from the different genotypes of two wheat varieties to our result, we first detected the m^6^A levels and mRNA expression of these selected genes in two healthy wheat variety plants. Then the results displayed that most genes had no significant changes either in m6A levels or mRNA levels between two varieties (Figures 5A,B). Subsequently, we performed m6A qPCR and normal RT-qPCR assays for these genes in WRV and WSV group and the results showed that vast majority of these genes (8/10) exhibited similar m^6^A-level changes, consistent with the m^6^A-seq data and demonstrating the reliability of our m^6^A-seq data (Figure 5C). Furthermore, normal RT-qPCR of these genes in three pairs of WRV samples and WSV samples was performed, and the results showed the same tendency to RNA-seq data (Figure 5D). Therefore, these results confirmed that not only the m^6^A-levels but also the mRNA levels of most genes all had similar tendencies to our seq-data.

Finally, we screened out many genes associated with plant resistance to pathogens through a series of bioinformatics analyses performed on the m6A-seq data and RNA-seq data. m6A qPCR also verified that eight of these genes indeed exhibited m^6^A methylation. In general, these results indicated that the differential m^6^A modifications between the two varieties may disturb these host-pathogen interaction pathways by regulating the expression of related genes, thereby affecting the virus infection process.

## Discussion

N^6^-methyladenosine methylation is the most prevalent post-transcriptional modification, which is extensively present in mRNA, tRNA, miRNA, and long non-coding RNA, and plays important roles in plant responses to various biotic and abiotic stresses (Cantara et al., 2011; Wei et al., 2017; Arribas-Hernández and Brodersen, 2020). Previous studies have shown that RNA viruses, especially mammalian RNA viruses, infecting host plants are N^6^-methylated to affect viral replication and infection (Tan and Gao, 2018; Tan et al., 2018; Hao et al., 2019). Nevertheless, it remains unclear whether plant RNA viruses can be N^6^-methylated after infection and whether such methylation can induce variations in transcriptome-wide m^6^A modification patterns to influence virus infection. In this study, we, for the first time, illustrated different global m^6^A modification patterns in two wheat varieties, with different resistances to WYMV and provided new insights into m^6^A modification regulation during WYMV infection.

To date, a large number of m^6^A transcriptome-wide analyses in plants have been carried out, with the development of m^6^A-seq. It has been reported that the m^6^A modification is not randomly distributed in RNA transcripts, but is mainly distributed in 3′-UTRs and stop codons, especially near the end of the 3′-terminal of CDS and the front of 3′-UTRs in plants (Batista, 2017; Yang et al., 2018). In the present study, more than 90% of the m^6^A peaks were found in 3′-UTRs and the stop codons of CDS (Figure 1C). We found that two high consensus motifs, UGUAY and GAACU, were significantly enriched in the m^6^A peaks identified in wheat (Figure 1D), similar to the observation in other plant species, such as rice and maize, in some previous studies (Li et al., 2014; Du et al., 2020). These results further demonstrated the conserved features of m^6^A methylation in plants.

The m^6^A modification has been proven to be a significant RNA modification, playing a critical role in various steps of mRNA function, including mRNA stability, degradation, and expression (Luo et al., 2014; Visvanathan and Somasundaram, 2018). In addition, there is also evidence that the m^6^A modification is involved in the regulation of biotic stress response and that different stresses can cause a transcriptome-wide redistribution of m^6^A (Yue et al., 2019). For example, AMV infection increases m^6^A levels in *Arabidopsis* (Martínez-Pérez et al., 2017). In the present study, conjoint analysis of m^6^A-seq and mRNA-seq data identified 729 genes in WRV that showed differences in both m^6^A levels and mRNA expression levels (Figure 4A). Half of these genes were negatively correlated with m^6^A modification, and the other half showed a positive correlation, which may be due to the different m^6^A modification regions in transcripts. Luo et al. (2014) found that m^6^A modifications in 3′-UTR and 5′-UTR regions are positively correlated with gene expression, while m^6^A modifications in other regions result in lower gene expression in Arabidopsis. Moreover, these genes are involved in many biological processes, such as protein phosphorylation, defense response, and the abscisic acid (ABA)-activated signaling pathway (Figures 4B,C). According to a previous study, phosphorylation and ABA are both closely related to plant-virus interactions (Alazem and Lin, 2017). For example, the Barley stripe mosaic virus γb protein can suppress RNA silencing and host cell death response *via* PKA-mediated phosphorylation to promote infection (Zhang et al., 2018). Therefore, we speculated that the genes involved in these pathways may perform important functions in the wheat-WYMV interaction-related pathways and are worthy of further investigation. Furthermore, the gene-specific m^6^A RT-qPCR verified the different m^6^A methylation modifications in TraesCS7B02G446900, TraesCS7A02G267400, TraesCS1B02G175900, TraesCS5B02G474500, TraesCS5B02G 447100, TraesCS7D02G341100, TraesCS1D02G381400, TraesCS2A02G312600, TraesCS1D02G176900, and TraesCS 2D02G321400 between the WRV and WSV groups, which may be due to the abnormal expression of key m^6^A enzymes. We found that one gene (TraesCS4D02G261802) encoding a writer protein TaFIP37-1 and another gene (TraesCS4D02G261812) encoding an eraser protein TaALKBH29B both exhibited abnormal expression according to our RNA-seq data (Supplementary Table 3). We detected the mRNA expression of these two genes in two healthy wheat variety plants and WYMV-infected two wheat variety plants, the results showed no significant changes in health group but there are obvious changes in WYMV infected group (Supplementary Figure 4). Therefore, our results inferred that TaFIP37-1 and TaALKBH29B may be involved in the different m^6^A modifications in the WRV and WSV groups, but further experiments are needed to confirm this result.

Studies have reported that *Oryza sativa* glucose−regulated protein 94 (*OsGRP94*), a homologous gene of TraesCS7B02G4 46900.1, is downregulated under dithiothreitol -induced endoplasmic reticulum stress, and thus OsGRP94 may participate in ER stress-induced autophagy and programmed cell death (Cui et al., 2016). Indeed, the HCV E2 protein can upregulate GRP94 expression to inhibit the apoptosis induced by HCV infection and the host immune system (Lee et al., 2008). The TraesCS1B02G175900.1 gene encodes a cysteine-rich receptor-like protein kinase, the homologous gene of which has been reported to be related to wheat resistance to stripe rust fungus (Zhou et al., 2007). TraesCS7A02G267400.1 encodes PTI1-like tyrosine protein kinase 3, and its homologous gene, PTI1-like tyrosine-protein kinase 1, which has been identified as a putative candidate resistant gene for soil-borne wheat mosaic virus (SBWMV) (Liu et al., 2020).

## Conclusion

In summary, the genes investigated in this study were all closely related to plant immunity and plant resistance to pathogens. Therefore, they can be used as candidate genes to explore the strategies of wheat resistance to viral infection, and to elucidate the mechanisms by which viruses successfully infect wheat. Further functional experiments are needed to verify the regulatory role of m^6^A RNA modifications in the expression of these candidate genes in plants against viral infection.

## Data Availability Statement

The datasets presented in this study can be found in online repositories. The name of the repository and accession number can be found below: National Center for Biotechnology Information (NCBI) BioProject, https://www.ncbi.nlm.nih.gov/bioproject/, PRJNA694346.

## Author Contributions

JY and JC conceived the project and designed the experiments. TZ and JY carried out the experiments with assistance from ZW, ZC, HH, PL, SG, FZ, LH, MX, and PJ. JY, TZ, and JC wrote the manuscript. All authors analyzed and discussed the results.

## Conflict of Interest

The authors declare that the research was conducted in the absence of any commercial or financial relationships that could be construed as a potential conflict of interest.

## Footnotes

1 https://github.com/OpenGene/fastp

2 http://daehwankimlab.github.io/hisat2

3 https://bioconductor.org/packages/exomePeak

4 http://www.igv.org

5 http://meme-suite.org

6 http://homer.ucsd.edu/homer/motif

7 https://ccb.jhu.edu/software/stringtie

8 https://bioconductor.org/packages/edgeR

## Supplementary Material

Supplementary Figure 1 RT-qPCR assays of WYMV CP in WRV and WSV samples.

Supplementary Figure 2 Unique m^6^A peak distribution in the indicated regions in WRV and WSV samples.

Supplementary Figure 3 The phenotype of two variety plants infected with WYMV.

Supplementary Figure 4 RT-qPCR assay of TaFIP37-1 and TaALKBH29B.

Supplementary Table 1 Statistics and quality control of raw data generated by sequencing.

Supplementary Table 2 Summary of the RNA-seq and m6A-seq of two variety.

Supplementary Table 3 List of the expressions of key m6A enzymes according to RNA-seq data.

Supplementary Table 4 The sequences of the primers used in these experiments.
