# Supplementary material for: Transcriptome-Wide N6-Methyladenosine (m6A) Profiling of Susceptible and Resistant Wheat Varieties Reveals the Involvement of Variety-Specific m6A Modification Involved in Virus-Host Interaction Pathways
Source: Front Microbiol. 2021 May 26;12:656302. doi: 10.3389/fmicb.2021.656302 (PMC8187603; doi:10.3389/fmicb.2021.656302)
Supplement: Supplementary Figure 1 — RT-qPCR assays of WYMV CP in WRV and WSV samples. [file Data_Sheet_1.docx]

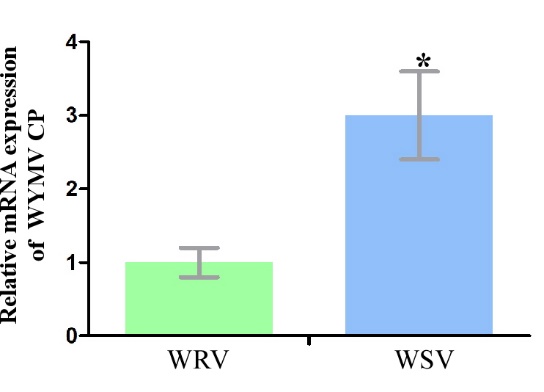


**Supplementary Fig. S1** **RT-qPCR assay of *WYMV CP* in WRV and WSV samples**

The level of *WYMV CP* in WRV group was normalized to 1. Each relative expression level is presented as the mean ± SD from three biological samples and each biological sample had three technical replicates. Statistical analyses were done using the Student’s *t*-test. Asterisks indicate a significant difference when compared to the control. * p < 0.05. WRV: WYMV-infected resistant wheat variety; WSV: WYMV-infected sensitive wheat variety.


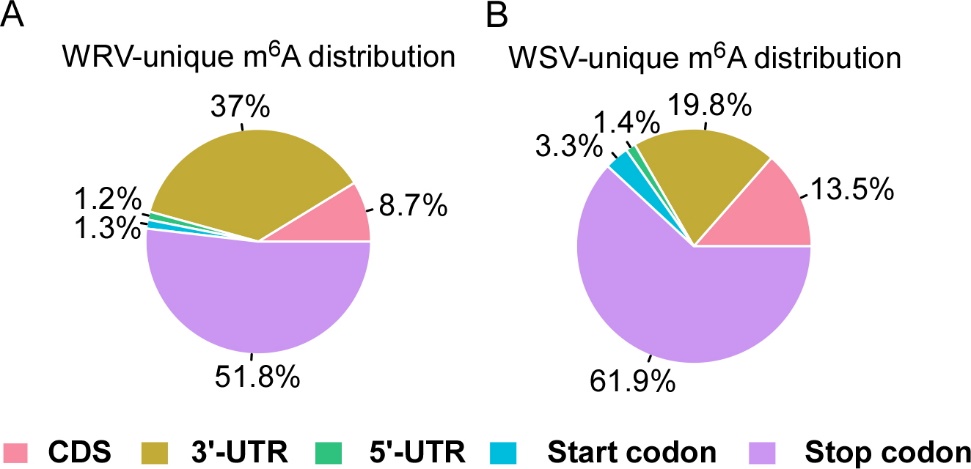


**Supplementary Fig. S2 Unique m^6^A peak distribution in the indicated regions in WRV and WSV samples**

1. WRV unique m^6^A peak distribution in different regions of the genome.

(b) WSV unique m^6^A peak distribution in different regions of the genome. WRV: WYMV-infected resistant wheat variety; WSV: WYMV-infected sensitive wheat variety


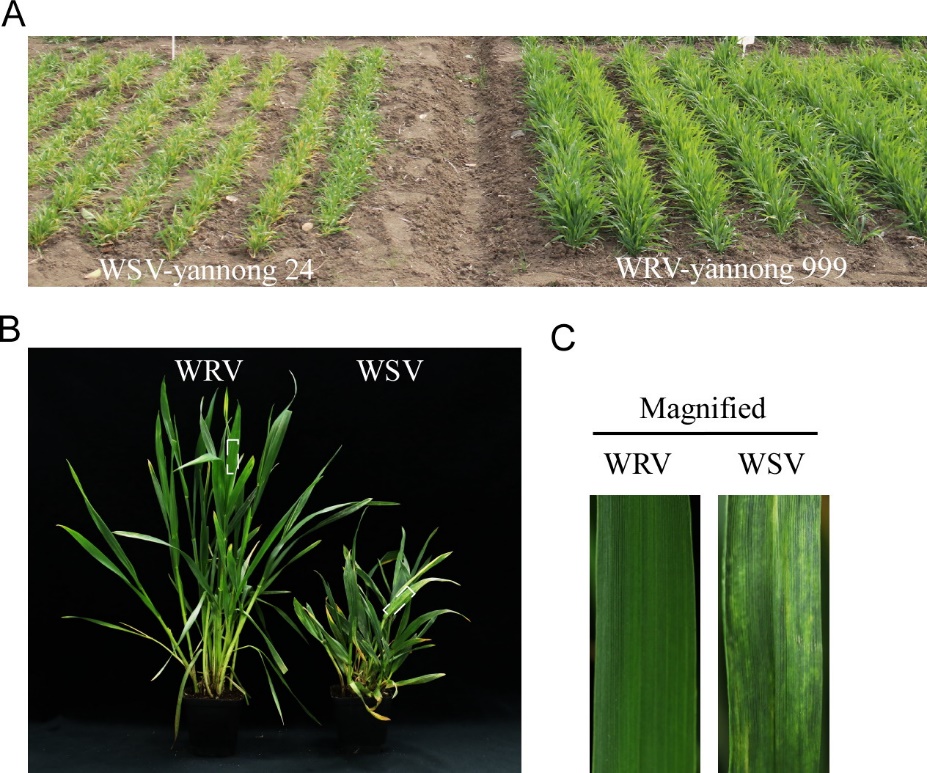


**Supplementary Fig. S3. The phenotype of two variety plants infected with WYMV** (A) WSV wheat plants (yannong24) showed obvious WYMV disease symptoms and WRV wheat plants (yannong999) showed no obvious symptoms in the field nursery. (B) Two typical wheat plant of two varieties from the field nursery infected with WYMV (C) Magnified wheat leaves in the dotted box in (B).


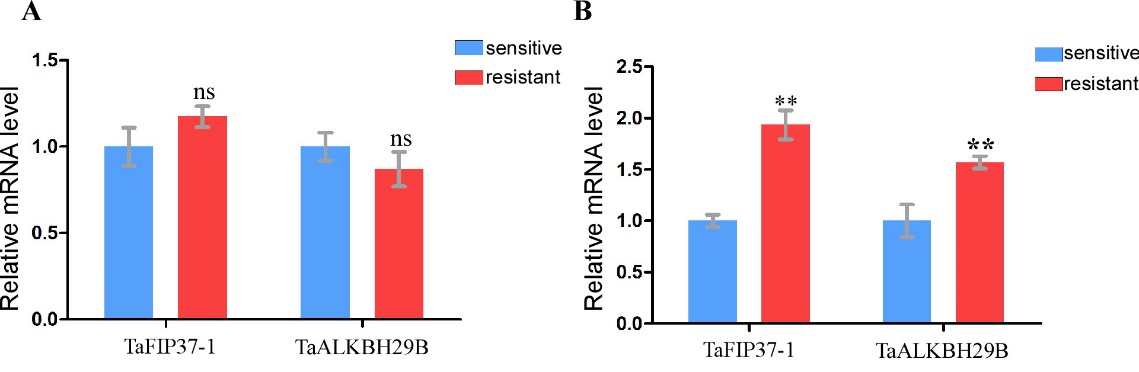


**Supplementary Fig. S4. RT-qPCR assay of** ***TaFIP37-1* and *TaALKBH29B.*** (A), (B) The mRNA levels of *TaFIP37-1 and TaALKBH29B* in two healthy groups and two WYMV infected groups (WRV, WSV) of sensitive variety and resistant variety. And the sensitive variety plants in two experiments was both normalized to 1. Each relative expression level is presented as the mean ± SD from three biological samples and each biological sample had three technical replicates. Statistical analyses were done using the Student’s *t*-test. Asterisks indicate a significant difference when compared to the control. * p < 0.05. WRV: WYMV-infected resistant wheat variety; WSV: WYMV-infected sensitive wheat variety.
