# Supplementary material for: Transcriptome-Wide N6-Methyladenosine (m6A) Profiling of Susceptible and Resistant Wheat Varieties Reveals the Involvement of Variety-Specific m6A Modification Involved in Virus-Host Interaction Pathways
Source: Front Microbiol. 2021 May 26;12:656302. doi: 10.3389/fmicb.2021.656302 (PMC8187603; doi:10.3389/fmicb.2021.656302)
Supplement: Supplementary Table 1 — Statistics and quality control of raw data generated by sequencing. [file Table_1.DOCX]

**Supplementary Table S1.** Statistics and quality control of raw data generated by sequencing.

| **Sample name** | **Raw Reads** | **Raw Bases** | **Valid Reads** | **Valid Bases** | **Q30%** | **GC%** |
| --- | --- | --- | --- | --- | --- | --- |
| WRV1_IP | 135310692 | 20.30G | 108779194 | 14.98G | 93.17 | 44.97 |
| WRV2_IP | 137411190 | 20.61G | 100482196 | 13.88G | 93.05 | 45.68 |
| WRV3_IP | 130689284 | 19.60G | 126298154 | 17.62G | 93.00 | 47.82 |
| WSV1_IP | 135724064 | 20.36G | 130830076 | 18.22G | 93.18 | 47.96 |
| WSV2_IP | 129797288 | 19.47G | 123965444 | 17.30G | 92.90 | 47.59 |
| WSV3_IP | 139808266 | 20.97G | 135278634 | 18.91G | 93.13 | 47.79 |
| WRV1_input | 129040482 | 19.36G | 126920716 | 17.80G | 94.62 | 49.75 |
| WRV2_input | 124788596 | 18.72G | 123147504 | 17.26G | 94.37 | 49.81 |
| WRV3_input | 135040572 | 20.26G | 132751752 | 18.57G | 94.32 | 50.89 |
| WSV1_input | 131341624 | 19.70G | 129383022 | 18.10G | 94.19 | 50.99 |
| WSV2_input | 136192916 | 20.43G | 133995650 | 18.73G | 94.08 | 51.03 |
| WSV3_input | 135417496 | 20.31G | 133466900 | 18.59G | 95.03 | 51.31 |

WRV: WYMV infected resistant wheat variety; WSV: WYMV infected sensitive wheat variety; Q30%: Quality Score≥30 (sequencing error rate less than 0.001).
