# Supplementary material for: Transcriptome-Wide N6-Methyladenosine (m6A) Profiling of Susceptible and Resistant Wheat Varieties Reveals the Involvement of Variety-Specific m6A Modification Involved in Virus-Host Interaction Pathways
Source: Front Microbiol. 2021 May 26;12:656302. doi: 10.3389/fmicb.2021.656302 (PMC8187603; doi:10.3389/fmicb.2021.656302)
Supplement: Supplementary Table 2 — Summary of the RNA-seq and m6A-seq of two variety. [file Table_2.DOCX]

**Supplementary Table S2.** Summary of the RNA-seq and m6A-seq of two variety.

| **Sample name** | **Seq Type** | **Total reads** | **Mapped Reads** | **Unique Mapped reads** | **Multi Mapped reads** |
| --- | --- | --- | --- | --- | --- |
| WRV_1 | m6A-seq (IP) | 107812842 | 101462027(94.11%) | 83010528(77.00%) | 18451499(17.11%) |
| WRV_2 |  | 100012188 | 91016080(91.00%) | 74395324(74.39%) | 16620756(16.62%) |
| WRV_3 |  | 126132050 | 119795928(94.98%) | 93503999(74.13%) | 26291929(20.84%) |
| WSV_1 |  | 130395312 | 124112458(95.18%) | 97610727(74.86%) | 26501731(20.32%) |
| WSV_2 |  | 123516294 | 117729136(95.31%) | 92826867(75.15%) | 24902269(20.16%) |
| WSV_3 |  | 134815312 | 128594124(95.39%) | 100563984(74.59%) | 28030140(20.79%) |
| WRV_1 | RNA-seq (Input) | 122502562 | 106716945(87.11%) | 82937788(67.70%) | 23779157(19.41%) |
| WRV_2 |  | 120549374 | 87887414(72.91%) | 68144945(56.53%) | 19742469(16.38%) |
| WRV_3 |  | 131656814 | 123078286(93.48%) | 93693816(71.17%) | 29384470(22.32%) |
| WSV_1 |  | 127447032 | 122031105(95.75%) | 90415772(70.94%) | 31615333(24.81%) |
| WSV_2 |  | 131311000 | 125682604(95.71%) | 92775130(70.65%) | 32907474(25.06%) |
| WSV_3 |  | 131190888 | 126376016(96.33%) | 93281805(71.10%) | 33094211(25.23%) |

RNA-seq: RNA-sequencing; m6A-seq: m6A-sequencing; WRV: WYMV infected resistant wheat variety; WSV: WYMV infected sensitive wheat variety.
