# Supplementary material for: Transcriptome-Wide N6-Methyladenosine (m6A) Profiling of Susceptible and Resistant Wheat Varieties Reveals the Involvement of Variety-Specific m6A Modification Involved in Virus-Host Interaction Pathways
Source: Front Microbiol. 2021 May 26;12:656302. doi: 10.3389/fmicb.2021.656302 (PMC8187603; doi:10.3389/fmicb.2021.656302)
Supplement: Supplementary Table 3 — List of the expressions of key m6A enzymes according to RNA-seq data. [file Table_3.DOCX]

**Supplementary Table S3.** List of the expressions of key m6A enzymes according to RNA-seq data.

| **Gene name** | **Gene Id** | **Chromosome** | **gene start** | **gene end** | **Log_2_ FC** | **P-value** |
| --- | --- | --- | --- | --- | --- | --- |
| TaMTA | TraesCS6A02G254900.1 | chr6A | 472504385 | 472509548 | -0.30821 | 0.2092 |
| TaMTC | TraesCS4D02G261800.1 | chr4D | 432764150 | 432767178 | -0.47708 | 0.2446 |
| TaMTB | TraesCS4D02G261801.1 | chr4A | 684583968 | 684589434 | -0.35212 | 0.1636 |
| TaFIP37-1 | TraesCS4D02G261802.1 | chr7D | 440041158 | 440044852 | 1.823835 | 0.0024 |
| TaFIP37-2 | TraesCS4D02G261803.1 | chr7D | 197297125 | 197302638 | -0.3342 | 0.1885 |
| TaVIR | TraesCS4D02G261804.1 | chr7A | 494770940 | 494787713 | -0.04559 | 0.6630 |
| TaHAKAI1 | TraesCS4D02G261805.1 | chr1D | 228301545 | 228304731 | -0.45594 | 0.1438 |
| TaECT3 | TraesCS4D02G261806.1 | chr7B | 625715946 | 625721126 | -0.23729 | 0.3540 |
| TaECT7 | TraesCS4D02G261807.1 | chr5D | 492726393 | 492730926 | 0.31431 | 0.5569 |
| TaECT6 | TraesCS4D02G261808.1 | chr5D | 492720111 | 492724223 | -0.24069 | 0.3218 |
| TaCPSF30 | TraesCS4D02G261809.1 | chr6A | 110348180 | 110352133 | -0.18797 | 0.6501 |
| TaALKBH4B | TraesCS4D02G261810.1 | chr7A | 46772176 | 46777567 | 0.024141 | 0.8411 |
| TaALKBH6B | TraesCS4D02G261811.1 | chr1B | 460273342 | 460281237 | 0.485823 | 0.3050 |
| TaALKBH29B | TraesCS4D02G261812.1 | chr4D | 390600705 | 390606786 | 1.41473 | 0.0026 |
